# Based on Mitochondrial Genomes and Gene Order Rearrangements: Phylogenetic Relationships and Terrestrial Adaptability in Paguroidea (Crustacea: Decapoda)

**DOI:** 10.1002/ece3.71975

**Published:** 2025-08-08

**Authors:** Zhengfei Wang, Zhixuan Wang, Xin Chen, Weijie Jiang, Chong Cui, Lijie Cui

**Affiliations:** ^1^ Jiangsu Key Laboratory for Bioresources of Saline Soils, Jiangsu Synthetic Innovation Center for Coastal Bio‐Agriculture, Jiangsu Provincial Key Laboratory of Coastal Wetland Bioresources and Environmental Protection, School of Wetlands Yancheng Teachers University Yancheng Jiangsu Province China; ^2^ College of Bioscience and Biotechnology Yangzhou University Yangzhou Jiangsu Province China; ^3^ Zhumadian Third Senior High School Zhumadian City Henan Province China; ^4^ College of Biotechnology and Pharmaceutical Engineering Nanjing Tech University Nanjing Jiangsu Province China

**Keywords:** Anomura, gene rearrangement, mitogenome, phylogeny, terrestrial adaptations

## Abstract

The complete mitochondrial genome provides pivotal information that enhances our understanding of molecular phylogenetic analysis, evolution, and gene rearrangement. Anomura, a decapod taxon with exceptional phenotypic diversity, inhabits hydrothermal vents and various aquatic and terrestrial habitats. However, debates regarding the deep‐level phylogeny of Anomura persist, particularly concerning its complex evolutionary relationships. Within this context, the superfamily Paguroidea emerges as a group of significant interest due to its unique biology and potential to illuminate broader anomuran evolutionary questions. Herein, we determined the details of mitogenomes in five Paguroidea species and further investigated phylogenetic relationships and divergence times of Anomura. Our study revealed that Paguroidea is paraphyletic, with its internal relationships still requiring further discussion. Additionally, phylogenetic analyses indicated that Coenobitidae diverged from aquatic Diogenidae and subsequently adapted to terrestrial habitats. Hence, we investigated its mechanisms of genetic rearrangement and conducted a foreground branch selection pressure analysis with Coenobitidae as the focal lineage. Branch‐site selection pressure analysis identified positive selection on *ATP6*, *ND3*, *ND2*, *ND5*, and *ND6*. Therefore, we hypothesized that Coenobitidae has increased its energy metabolism through the evolution of these genes, which may be advantageous for its adaptation to terrestrial environments. Our findings provide valuable insights into the evolution of Anomura species and offer a theoretical basis for the conservation and utilization of Anomura genetic resources.

## Graphical abstract

Investigating the internal phylogenetic relationships within Paguroidea and the terrestrial adaptability of Coenobitidae within Paguroidea through the analysis of mitochondrial genomes of five Anomura species.

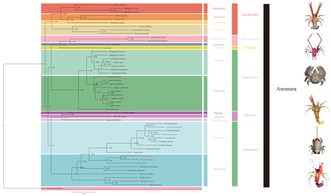

## 1 Introduction

As a key group of Decapod crustaceans, the infraorder Anomura is distinguished by its unique morphological traits and ecological diversity (Wolfe et al. 2019). Among these species, hermit crabs represent the most recognized members of the infraorder Anomura, inhabiting a wide range of aquatic habitats encompassing shallow littoral zones, diverse marine ecosystems (such as deep‐sea hydrothermal vent communities and hadal zones), as well as terrestrial environments (Tan et al. 2018). Their ecological adaptability and biodiversity make them integral to marine ecosystems, as well as the exotic pet trade (Calado et al. 2003). Furthermore, species like crayfish hold potential as viable models for the investigation of cancer and aging processes (Vogt 2018). Studying Anomura is essential for understanding biodiversity, ecological functions, and the sustainable management of marine resources (Chaves et al. 2019).

Over the past decades, numerous studies have been conducted on the morphological and ecological characteristics of Anomura, which are indeed pivotal in species identification and classification, with traits such as carapace structure, appendage morphology, and coloration patterns (Diawol et al. 2021). However, these studies often struggle to resolve deeper phylogenetic relationships and may fail to capture cryptic diversity within these genera (Hamasaki et al. 2017). Recent advances in genetic sequencing techniques have enabled significant progress in resolving phylogenetic uncertainties (Gong, Lu, et al. 2019). Studies utilizing mitochondrial markers (Sziszkosz et al. 2016), such as *COX1* and *16S rRNA*, as well as nuclear genes (Yuan et al. 2017), have successfully clarified previously unresolved phylogenetic relationships, adaptive evolution, and genetic diversity problems (Bracken et al. 2010). Nonetheless, the phylogenetic relationships within the Paguroidea (Anomura) remain a subject of debate (Wang et al. 2024). Addressing these gaps requires comprehensive, multi‐disciplinary studies that integrate advanced molecular techniques (Mejías‐Alpízar et al. 2024), such as whole‐genome sequencing and transcriptomics, with traditional morphological analyses and ecological modeling (Zimmermann et al. 2016).

The superfamily Paguroidea includes the family Coenobitidae, a unique group of terrestrial hermit crabs that have evolved remarkable adaptations for life on land. However, in this family, *Coenobita* still relies on the marine environment for larval development, a trait also observed in other species of marine hermit crabs (Gong, Lu, et al. 2019). Conversely, *Calcinus* species, or coral hermit crabs, are associated with coral reef ecosystems and display a range of morphological and ecological specializations (Gong, Jiang, et al. 2019). These two genera provide unique opportunities to study evolutionary adaptation, ecological roles, and biodiversity within marine and semi‐terrestrial systems (Hyžný et al. 2016). Despite their ecological importance, the genetic and evolutionary relationships among these species remain insufficiently explored (Machordom et al. 2022).

Herein, we newly sequenced the five complete mitochondrial genomes of Anomura (*Coenobita violascens*, *Coenobita purpureus*, *Calcinus elegans* , *Calcinus gaimardii* , and *Calcinus latens* ) and reconstructed comprehensive phylogenetic and divergence time trees to further explore the taxonomic position and internal relationships within Paguroidea. Additionally, to investigate the terrestrial adaptability of *Coenobita*, we assessed the selection pressure on mitochondrial OXPHOS genes specifically. These genes under positive selection may suggest that *Coenobita* requires greater energy to adapt to terrestrial habitats. Furthermore, the potential genetic rearrangement mechanisms across diverse Paguroidea species were re‐investigated to elucidate the underlying evolutionary processes of this intricate group. These research outcomes will contribute to a more nuanced understanding of Anomura biodiversity and lay a solid foundation for subsequent phylogenetic studies of Anomura.

## 2 Materials and Methods

### 2.1 Samples and DNA Extraction

In this study, five specimens from two families (three from Coenobitidae, and two from Diogenidae) were sourced from Sanya (Hainan, China) (Figure S1). Muscle tissues were dissected, rapidly frozen in liquid nitrogen, and stored at −80°C. Total DNA was extracted from the muscle tissue of each sample using the Aidlab Genomic DNA Extraction Kit (Aidlab Biotech, Beijing, China), and promptly placed in 1 × TAE buffer at 4°C for temporary storage. Subsequently, we assessed the quality of the DNA using 1.5% agarose gel electrophoresis and, upon confirmation of its integrity, transferred it to storage at −20°C.

### 2.2 Sequence Assembly, Annotation, and Analysis

The amplification of mitochondrial sequences was conducted using universal primers, including *16S rRNA* and *COX1* (Tang et al. 2020) (Table S1). The PCR reactions were carried out on an ABI 9700 DNA amplifier, with a reaction mixture consisting of 12.5 μL of 2 × F8 PCR MasterMix, 0.5 μL of each reverse and forward primer, 1.5 μL of DNA template, and 10 μL of ddH_2_O. To ascertain sequence homology of the assembled mitochondrial genomes with known species, *16S rRNA* and *COX1* barcode sequences were analyzed utilizing the comparative search functionality of BLAST in NCBI. New species were sequenced by next‐generation sequencing (Majorbio Bio‐Pharm Technology Co. Ltd., Shanghai, China), and each species yielded 10G raw reads (coverage 3–5×). Quality‐filtered reads were subsequently assembled using Geneious Prime 2024.0 software, with reference mapping performed against the complete set of 37 mitochondrial genes from the closest available relative in NCBI databases. The mitogenomic data for the five newly discovered species were subsequently deposited in the GenBank database (GenBank: PV126463‐PV126467).

The initial determination of the approximate gene boundaries was conducted using MITOS2 (http://mitos2.bioinf.uni‐leipzig.de/index.py) (Kawashima et al. 2013), with specific settings designated for Metazoa: 89 and invertebrate: 5. In addition, MITOS2 and tRNAscan‐SE Web Server (https://lowelab.ucsc.edu/tRNAscan‐SE/) were employed to ascertain the cloverleaf secondary structure of tRNAs (Schattner et al. 2005). Furthermore, we utilized MEGA 11 to determine the nucleotide composition and applied the following formulas to assess the compositional skew: AT‐skew = (A − T)/(A + T) and GC‐skew = (G − C)/(G + C) (Tamura et al. 2011). The mitochondrial genome maps were constructed using the CGView Server (https://cgview.ca/) (Grant and Stothard 2008). The calculation of codon usage count and relative synonymous codon usage (RSCU) values for each protein‐coding gene (PCG) was performed using MEGA 11, followed by the elimination of incomplete stop codons (Tamura et al. 2011). The tandem repeats in the control region were detected using Tandem Repeats Finder 4.09 (https://tandem.bu.edu/trf/home) (Benson 1999). Additionally, genetic distances between and within species were calculated with MEGA 11 (Tamura et al. 2011).

### 2.3 Phylogenetic Analysis and Divergence Time Estimation

The nucleotide sequences for 47 mitogenomes, including 46 from Anomura and 1 from Brachyura, were retrieved from GenBank (Table S2). Together with the five newly sequenced species, these sequences were aligned using MUSCLE 3.8 within the MEGA software (Tamura et al. 2011). The 13 PCGs were then concatenated for further analysis. Phylogenetic analyses were conducted using MrBayes version 3.2.6 (Huelsenbeck and Ronquist 2001) and IQ‐tree (Nguyen et al. 2015), with the resulting trees visualized in FigTree version 1.4.2 (De Bruyn et al. 2014).

Divergence times were estimated based on nucleotide sequences of USCOs by using MCMCTree in PAML (Ziheng 2007). It estimates the divergence times of species trees using Bayesian methods. The fossil calibration points used for constructing divergence times are shown in Table S3, and the results of divergence time estimation were viewed using FigTree version 1.4.2 (De Bruyn et al. 2014).

### 2.4 Gene Rearrangement

To standardize the annotation outcomes and ensure uniformity across the mitogenomes of Anomuran species examined in this study, a comprehensive re‐annotation process was conducted using MITOS (Kawashima et al. 2013) and NCBI (Wang 2016), focusing on resolving any inconsistencies observed. Any discrepancies in the mitogenome annotations were rectified manually to guarantee the comparability, consistency, and precision of the data. The mitogenome sequences were subsequently aligned with MitoPhAST v2.0 (https://github.com/mht85/MitoPhAST) to aggregate homologous mitochondrial gene order (MGO) clusters (Tan et al. 2015). Furthermore, the Common Interval Rearrangement Explorer (CREx) (Bernt et al. 2007) or TreeRE (Bernt et al. 2008) was employed to delineate the evolutionary pathways of gene rearrangements, as well as to infer ancestral gene sequences and interspecies relationships among the organisms under investigation.

### 2.5 Select Pressure Analysis

The ratio of nonsynonymous to synonymous substitutions (*ω* = dN/dS) is a metric employed to uncover the evolutionary dynamics of genes and serves as a valuable tool for assessing the impact of selection pressures on molecular evolution. Specifically, values of *ω* > 1, = 1, and < 1 signify positive selection, neutral evolution, and purifying selection, respectively (Reyes et al. 1998). The CODEML module within the PAML v4.7 package was applied to identify instances of positive selection within PCGs (Yang and Nielsen 2000). A branch‐site model was employed to assess positive selection across various lineages within the superfamily Paguroidea. Bayesian Empirical Bayes (BEB) analysis was conducted to pinpoint sites under positive selection within the branch‐site model, with a probability threshold of 0.8. Additionally, to validate the findings from the PAML analysis, a complementary protein‐level approach was utilized (Guo et al. 2018), which involved the deployment of TreeSAAP v3.2 to identify alterations in the physicochemical characteristics of amino acids (Woolley et al. 2003).

## 3 Result

### 3.1 Characterization of Mitogenomes of the Five Species

**FIGURE 1:**
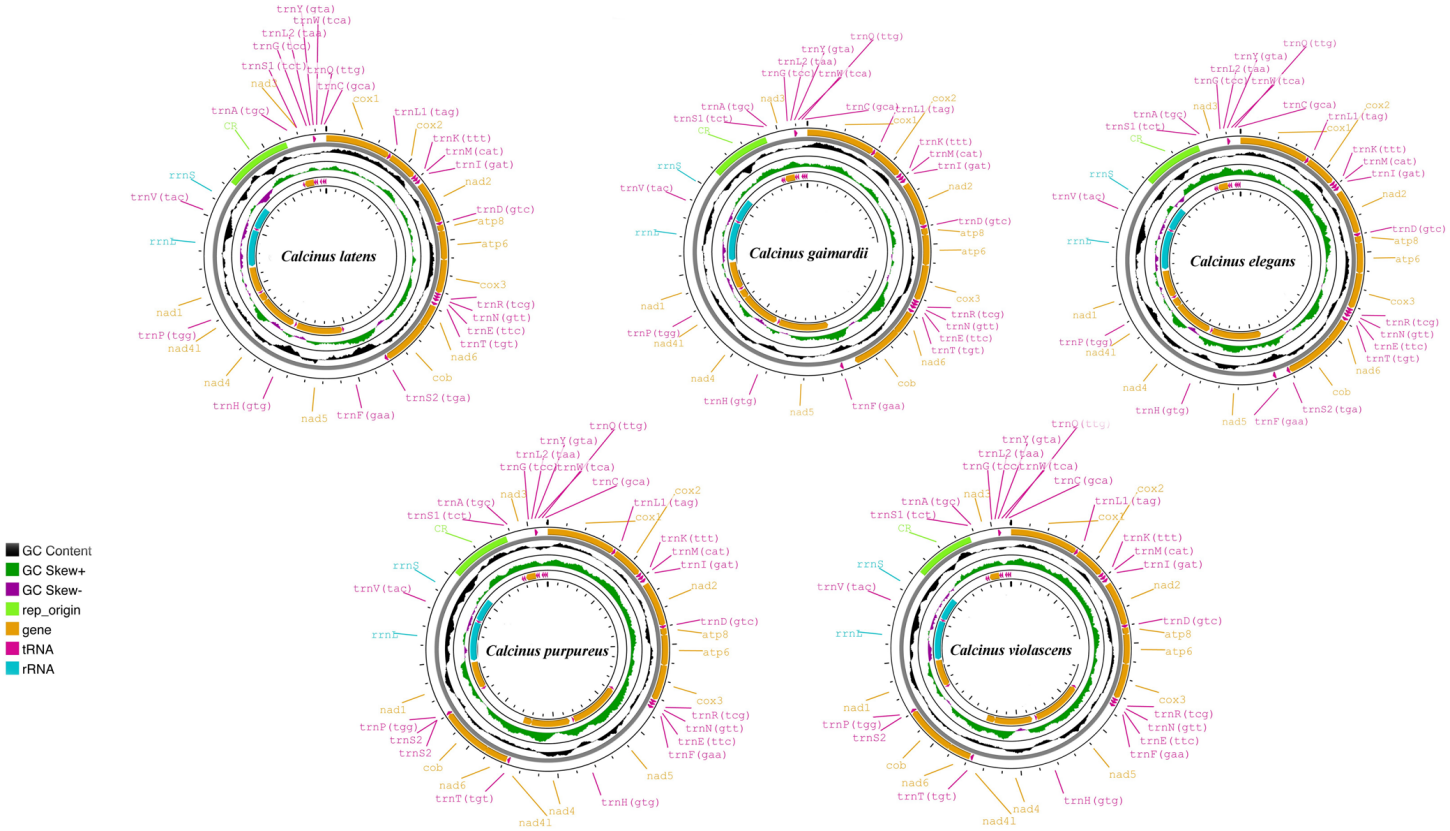
Complete mitochondrial genomes of five species of Anomura crabs: *Calcinus elegans* , *Calcinus gaimardii* , *Calcinus latens* , *Coenobita purpureus*, and *Coenobita violascens*.

Similar to the mitochondrial genomes of most metazoans, the mitogenomes of the five newly sequenced species are double‐stranded circular molecules, mostly encoding 13 protein‐coding genes (PCGs), 22 transfer RNA genes (tRNAs), two ribosomal RNA genes (*rrns* and *rrnl*), and the assumed non‐coding region (control region, CR) (Ruan et al. 2020). The lengths of these mitogenomes ranged from 16,421 to 17,445 bp, which fall within the range of mitochondrial genome sizes observed in the Anomura (from *Pagurus lanuginosus* with 13,458 bp to *Diogenes edwardsii* with 19,858 bp) (Gong et al. 2020) (Figure 1 and Table S4). The genes *ND5*, *ND4*, *ND4L*, *ND1*, and *ND3* were located in the light strand (L‐strand), while the remaining PCGs were located in the heavy strand (H‐strand) as indicated in Figure 1.

Intergenic spacers and overlapped regions were commonly found in these mitogenomes, among which the largest gene overlap region, spanning 20 bp, was observed between the *ND6* and *CYTB* genes in five species. Additionally, the AT content of the five species was relatively high, ranging from 62.05% in *C. purpureus* to 68.23% in *C. gaimardii* , which is likely the characteristic found in most metazoans. All five species exhibited negative AT skew and positive GC skew, indicating a preference for Ts and Gs over As and Cs in the base composition (Figure S2 and Table S5).

### 3.2 PCGs and Codon Usage

The length of PCG regions ranged from 11,184 bp in *C. violascens* to 11,190 bp in Calcinus (Table S5). Consistent with the norm for invertebrate mitogenomes, all protein‐coding genes (PCGs) across the five species have typical start codons for ATN (Zhang et al. 2023). The *CYTB* and *ND5* genes of all five species terminate with an incomplete T. Additionally, the *ND4* gene in *C. gaimardii* and the *ND2* gene in both Coenobita species also end with an incomplete T. In calculating relative synonymous codon usage (RSCU) values for the five species, the codons AGA, UUA, AGA, UCU, and UCU were utilized most prominently, exhibiting a pronounced bias towards A/U in the majority of codon usage (Figure S3). This A/U bias in codon selection could be attributed to factors such as gene expression levels and gene length, the secondary structure of mRNAs, and the repertoire of available tRNAs (Wu et al. 2022). Furthermore, variations in codon usage may mirror the origin, mutational profiles, and evolutionary history of species (Loretán et al. 2019).

### 3.3 tRNAs and rRNAs

The analysis of tRNAs and rRNAs in five species revealed that most species possess a typical set of 22 tRNAs (Figure S4), except for *C. gaimardii* , which lacks the *trnS2*. Most tRNAs displayed the expected cloverleaf structure, including four loops (the dihydrouridine [DHU] loop, the anticodon loop, the additional loop, and the TψC loop) and four stems (the amino‐acid acceptor stem, the DHU stem, the anticodon stem, and the TψC stem) (Dong et al. 2024). However, *trnI* in *C. elegans* and *C. latens* was missing one loop. Additionally, *trnS1* in all five species lacked the DHU loop, which is probably a common feature in these species. Interestingly, some mismatched pairings (such as G‐U, U‐U, and A‐C) were observed. Previous research suggests that these mismatches have minimal impact on the stability of the tRNA secondary structure (Zhang et al. 2024).

### 3.4 Control Region

The CR in the mitochondrial genome is extensively employed as a phylogenetic marker in invertebrates (Tolve et al. 2024). Typically, this segment is the sole notable part of the mitogenome that does not code for any recognized functional genes. Four control region motifs were reported in some arthropod mitogenomes, including tandem repeats, one poly‐T region, a stem‐loop structure, etc. (Cook 2005).

**FIGURE 2:**
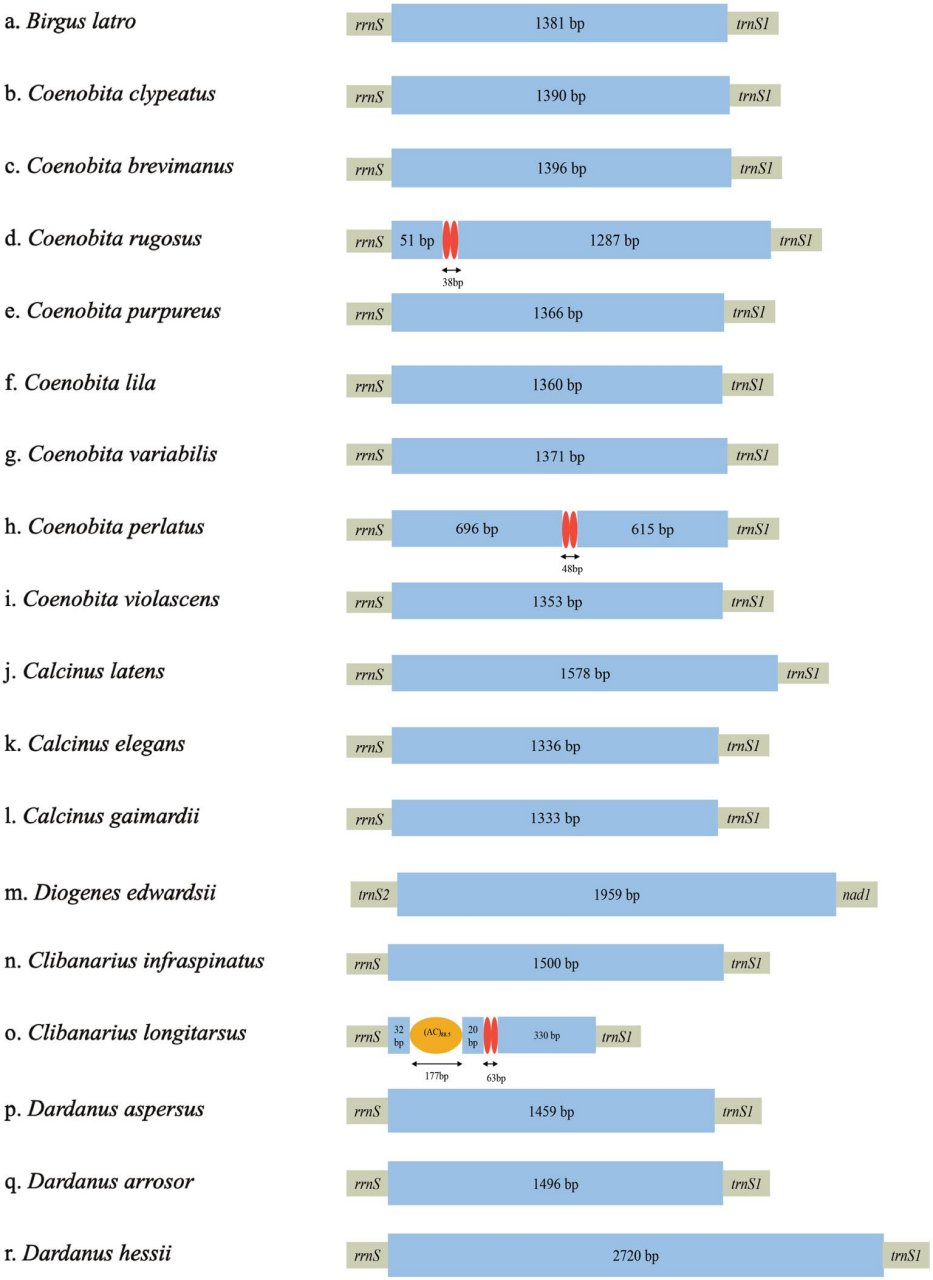
Structures of the control region in 16 Anomura mitogenomes. The colored ovals indicate the tandem repeats; the remaining regions are shown with blue boxes. The tandem repeats with a copy number exceeding 7 are displayed in the format of (motif) *n*.

Consistent with the mitogenomes of other Anomura crabs, the variation in mitochondrial genome sizes among the five species within Paguroidea is primarily attributed to differences in the size of the CR. For these five species, no tandem repeats were identified in their CRs, and all CRs are situated between the *rrnS* and *trnS1* genes (Figure 2). The CRs of these species exhibited a high A + T content, with the CR of *C. gaimardii* having the highest A + T content at 71.42%, with nucleotide compositions of 36.98% A, 34.43% T, 11.93% C, and 16.65% G. *C. latens*, in comparison to the other species, possessed a longer CR, which also accounts for its longer mitochondrial genome length (Table S5).

### 3.5 Skewness and Genetic Distance

**FIGURE 3:**
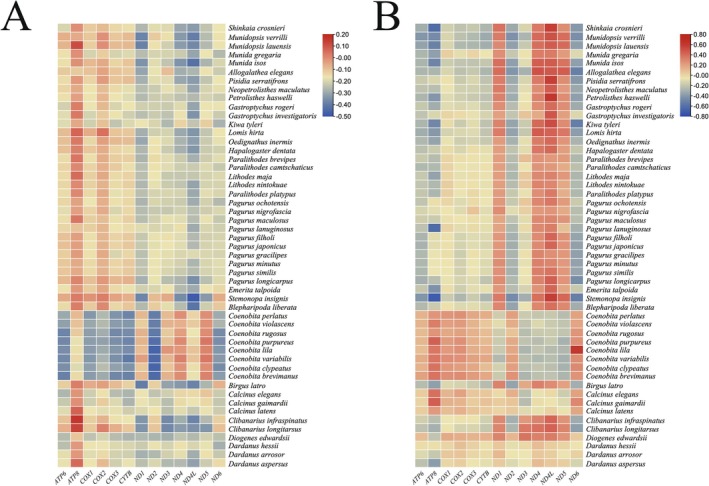
The AT content of the 52 species (A) and the GC content of the 52 species (B).

We calculate the nucleotide composition of mitogenomes in 52 species, of which species within the same genus tended to have consistent AT and GC skewness, and the sequence structures of species within the same genus have a certain degree of similarity, probably helpful to the classification to some extent (Xin et al. 2023). *C. infraspinatus* and *C. longitarsus* have a higher AT skew in *ATP8* compared to other species. *P. haswelli* has the highest GC skew in *ND4L* compared to other species (Figure 3, Tables S6 and S7).

**FIGURE 4:**
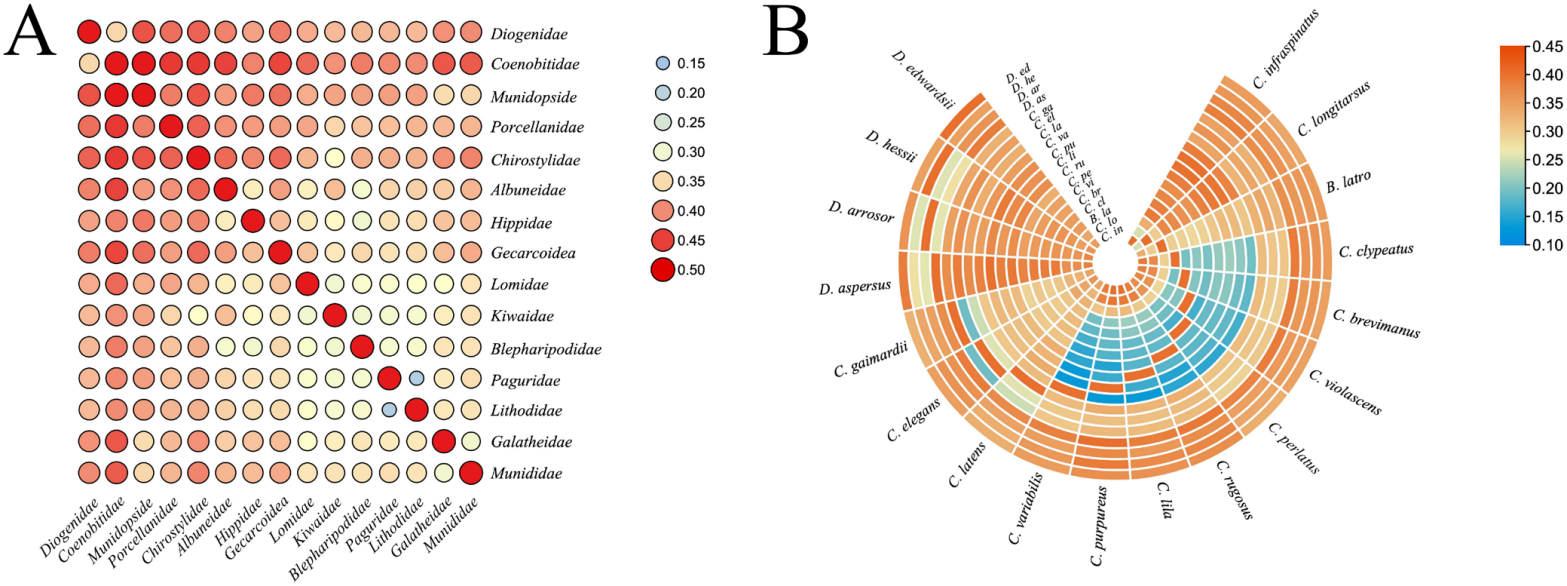
Genetic distance between 5 families (A) and genetic distance within the Coenobitidae and Diogenidae (B).

Among the genetic distances in 15 families, the biggest genetic distance was observed between Coenobitidae and Munidopsidae (Figure 4A and Table S8). Within the genetic distances of Coenobitidae and Diogenidae, the biggest genetic distance is found between *C. purpureus* and *Clibanarius infraspinatus* (Figure 4B and Table S8). This finding aligns with the phylogenetic tree, probably due to more distant relationships and increasing genetic divergence (Zhang et al. 2024).

### 3.6 Phylogenetic Analysis

**FIGURE 5:**
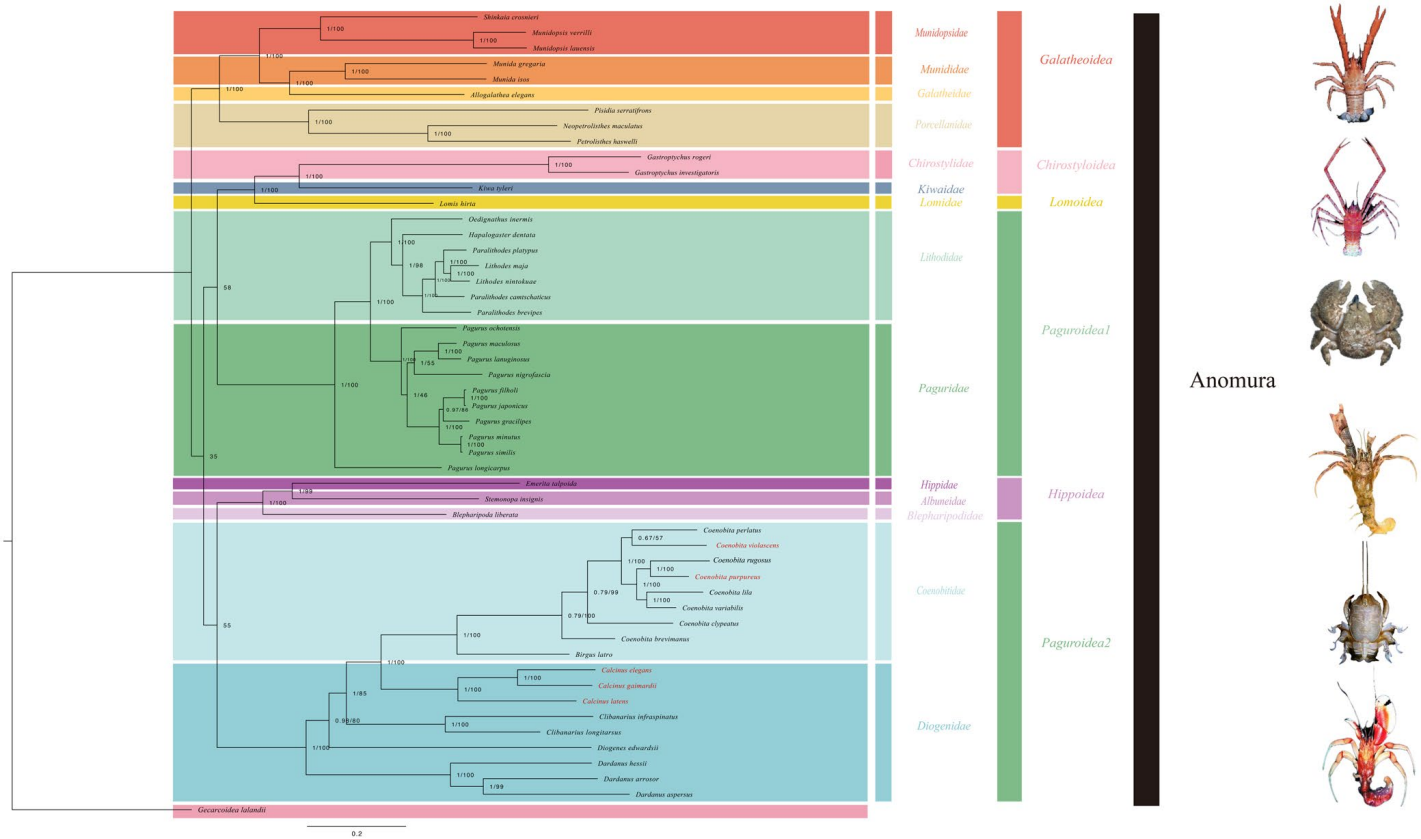
Phylogenetic trees were constructed from a dataset of 52 species using BI and ML methods. Species names highlighted in red are newly sequenced for this study.

The topological structure of the phylogenetic tree was reconstructed using the ML and BI methods, along with the nucleotide and protein sequences of 13 concatenated PCGs. The two phylogenetic trees were almost consistent; virtually all the key branches had high nodal support values (ML/BPP = 100/1.00). Therefore, only the ML topology was manifested, while the ML bootstrap values and BI posterior probabilities were displayed (Figure 5).

**FIGURE 6:**
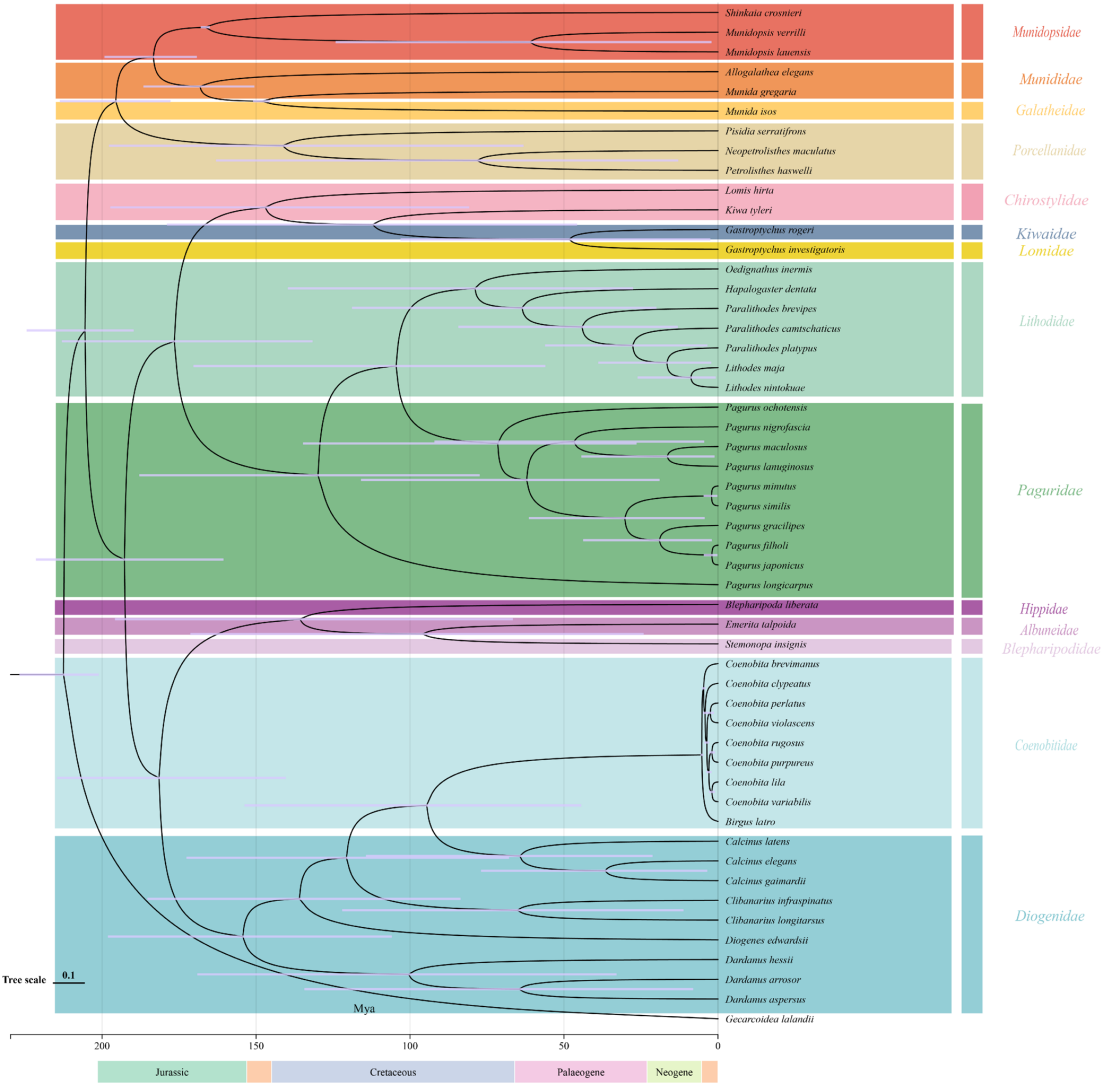
Divergence time estimation of 52 species derived from MCMCTree analysis. Numbers at nodes indicate estimated age. Purple bars represent 95% credible age intervals for each node.

Divergence times estimated the origin of the Anomura in the Triassic (~212 MYA, 95% credibility interval = 201–227 MYA, Figure 6). Within the Anomura, five long‐recognized superfamily groups were recovered: (Galatheoidea + ((Paguroidea1 + (Chirostyloidea + Lomoidea)) + (Hippoidea + Paguroidea))). According to the structure of the phylogenetic tree, species were categorized into two clades at the familial level: Clade 1: (((Munididae + Galatheidae) + Munidopsidae) + Porcellanidae); Clade 2: ((((Chirostylidae + Kiwaidae) + Lomidae) + (Lithodidae + Paguridae)) + (((Hippidae + Albuneidae) + Blepharipodidae) + (Diogenidae + Coenobitidae))). The monophyly of the majority of Anomura superfamily groups was strongly supported, except for Paguroidea, which was identified as a polyphyletic group. The phylogenetic tree revealed that Paguroidea exhibited paraphyly, with Paguroidea being divided into two clades: Paguroidea 1: (Paguridae + Lithodidae), and Paguroidea 2: (Coenobitidae + Diogenidae). These two clades are not inferred to share a common ancestor. The divergence time for Paguroidea1 is estimated to be ~130 MYA, while that for Paguroidea2 is estimated to be ~154 MYA (Figures 5 and 6).

Within the branch of Paguroidea 1, *Pagurus longicarpus* was observed to have first appeared ~130 MYA. Subsequently, Lithodidae and Paguridae diverged around 104 MYA. All species of Paguridae within Paguroidea 1, except for *Pagurus nigrofascia*, *Pagurus gracilipes*, and *Pagurus ochotensis* , were found to be paired sister groups (Figures 5 and 6). Paguroidea 2 was divided into two families, Coenobitidae and Diogenidae, with our study strongly supporting the monophyly of Coenobitidae. It is noteworthy that both the phylogenetic tree and divergence times indicated that the terrestrial hermit crabs of the family Coenobitidae evolved directly from the marine hermit crabs of the family Diogenidae, with a divergence time of ~53 MYA. The internal relationships within Diogenidae show a certain degree of paraphyly, with the three newly sequenced Diogenidae species in our study being more closely related to Coenobitidae and sharing a common ancestor. The branch (((Munididae + Galatheidae) + Munidopsidae) + Porcellanidae) was classified as Galatheoidea (~205 MYA), while the branches Chirostylidae and Kiwaidae formed a sister group (Figures 5 and 6).

### 3.7 Gene Rearrangement Among Paguroidea

Mitochondrial gene rearrangement serves as a pivotal molecular marker in evolutionary research, utilized as a significant analytical tool (Su et al. 2018). Herein, with 51 Anomura mitochondrial genomes analyzed, a total of 32 distinct types of MGOs (mitochondrial gene orders) were identified. Except for a minority of species exhibiting identical MGOs, the majority of Anomura species, including those within the same family or genus, display distinctly diverse MGOs, as observed in species belonging to the Munidopsidae family and the genus *Munida*. Particular attention was given to the MGOs (Patterns 27 through 32) within the species of Paguroidea 2 in this study (Figure S5 and Table S9).

**FIGURE 7:**
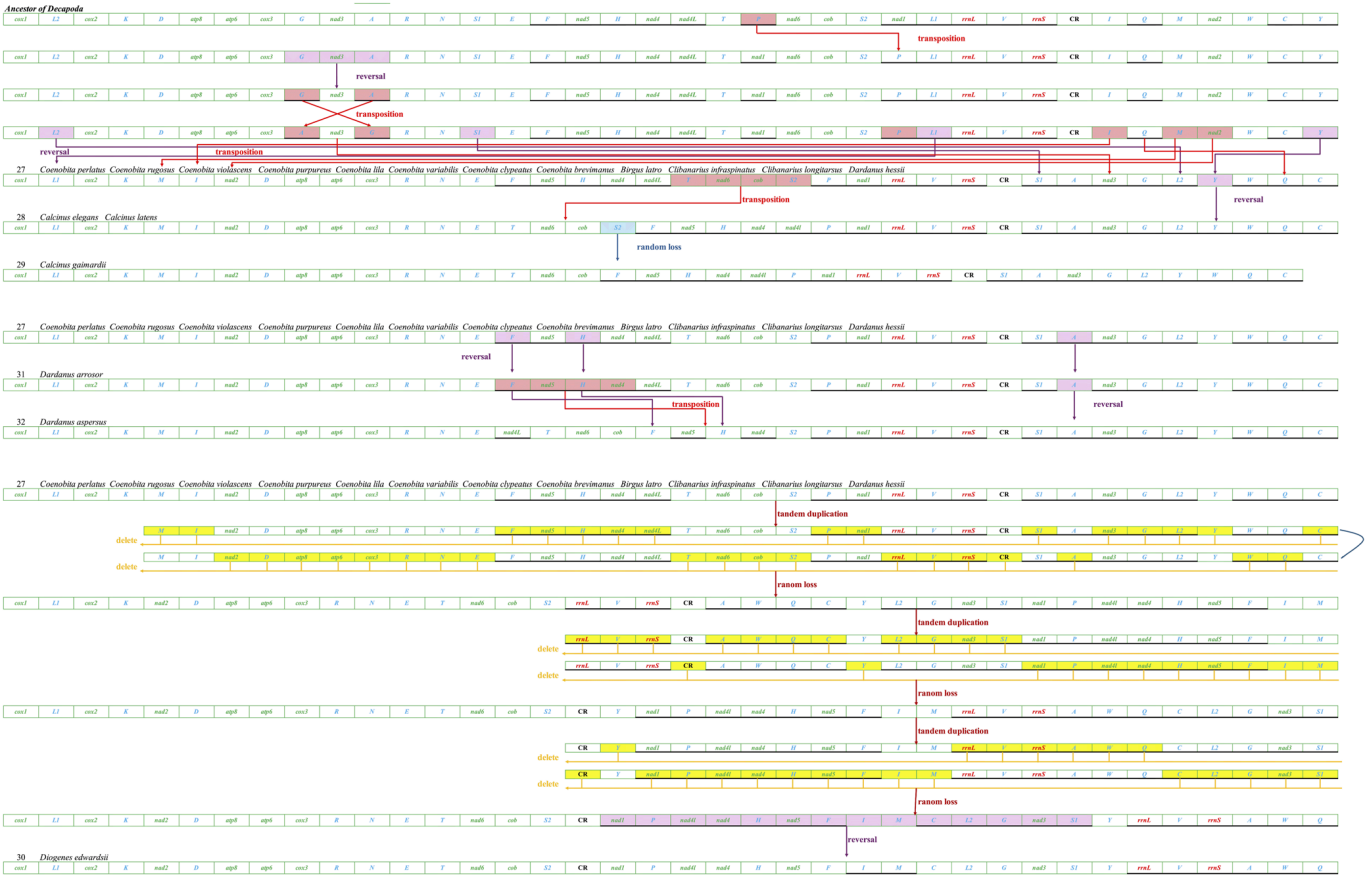
Schematic diagram of gene rearrangement mechanism in Paguroidea 2.

The mitochondrial gene order of the Ancestor of Decapoda underwent extensive rearrangement events, including multiple gene reversals and transpositions, ultimately evolving into Pattern 27. Among the 32 mitochondrial gene order (MGO) types identified, Pattern 27 (characterized by the segment “*T‐ND6‐CYTB‐S2*”) was conserved in Coenobitidae. In Pattern 27, the segment *T‐ND6‐CYTB‐S2* evolved into Pattern 28 through a single transposition with *F‐ND5‐H‐ND4* and a reversal of *trnF*, which subsequently lost *trnS2* to form Pattern 29 (Figure 7). By examining the rearrangement evolution diagram, it can be observed that the MGO of patterns 28 and 29 was highly similar to that of pattern 27. This further validated the closer relationship of the three newly sequenced Diogenidae species to Coenobitidae.

Pattern 31 was derived from Pattern 27 by the transposition of *trnF*, *trnH*, and *trnA* from the L‐strand to the H‐strand without any change in their relative positions. Pattern 32 differed from Pattern 31, with the transposition of *F‐ND5‐H‐ND4* to downstream of *ND4L‐T‐ND6‐CYTB*, and the inversion of *trnF*, *trnH*, and *trnA* from the H‐strand reversal to the L‐strand (Figure 7). Among the more complex changes within Paguroidea 2 was the evolution from Pattern 27 to Pattern 30. Pattern 27 experienced three TDRL events, which led to the evolution of large segments of the mitochondrial genome. Following these TDRL events, a reversal of the gene cluster *ND1‐P‐ND4L‐ND4‐H‐ND5‐F‐I‐M‐C‐L2‐G‐ND3‐S1* occurred without a shift in its position, culminating in the evolution to Pattern 30 (Figure 7).

### 3.8 Selection Pressure of PCGs

The *K*_a_/*K*_s_ ratio, which represents the relative frequency of non‐synonymous (*K*_a_) to synonymous (*K*_s_) nucleotide substitutions within coding sequences of genes, is a metric used in the field of molecular evolutionary biology to gauge the intensity and direction of selective pressures influencing protein‐coding genes (PCGs) (Hurst 2002). Saccone et al. (1999) determined that among mitochondrial genes in the majority of metazoans, *ATP8* exhibits the highest level of variability, followed by *ATP6*, *ND1*, *ND2*, *ND3*, *ND4*, *ND4L*, *ND5*, and *ND6* in terms of subsequent variability. In contrast, *CYTB*, *COX1*, *COX2*, and *COX3* were identified as more conserved mitochondrial genes.

Coenobitidae has fully adapted to terrestrial life. In contrast, Diogenidae, which is more closely related to Coenobitidae, still relies on marine habitats. Therefore, in the selection pressure analysis of Anomura, the monophyletic lineage of Coenobitidae was considered the foreground branch, and it was found that *ATP6*, *ND3*, *ND2*, *ND5*, and *ND6* underwent positive selection (Table S10). These genes under positive selection may indicate that Coenobitidae requires more energy to adapt to terrestrial habitats, and further analysis will be conducted in the discussion section.

## 4 Discussion

The mitochondrial genomic dataset employed in this study has furnished a more robust and encompassing phylogenetic tree and temporal framework for delineating the terrestrial incursion and evolutionary trajectories of crab species. The divergence dating analysis conducted has estimated the origin of the Anomura to be in the Triassic period (~212 MYA, 95% credibility interval = 201–227 MYA, Figure 6), originating from a symmetrical crab‐like ancestor. This estimation aligned broadly with prior research that, based on divergence time, suggested the origin of Anomura to be in the Late Permian, around 259 MYA (224–296 MYA) (Bracken‐Grissom et al. 2013). Our results in phylogenetic relationships were found to be largely consistent with those of previous studies (Tsang et al. 2008); (Wang et al. 2024). It was revealed that Anomura and Brachyura were sister taxa, with the hermit crab superfamily, Paguroidea, which is of interest to us, being recognized as paraphyletic. Therefore, the internal relationships within Paguroidea remain to be further discussed.

Hermit crabs are suggested to have diverged into two distinct groups, Paguroidea 1 and Paguroidea 2, during the early Cretaceous and late Jurassic periods (130 MYA and 154 MYA), supporting the polyphyletic origin of hermit crabs (Lü et al. 2023). Diogenidae, Coenobitidae, and Paguridae typically exhibit asymmetrical chelae, characterized by an enlarged right or left claw, a departure from the previously symmetrical form. Our analysis posited that the evolution of asymmetrical chelae occurred independently in two hermit crab lineages, once in Paguridae and another time in Diogenidae and Coenobitidae, a finding that diverged from prior research (Bracken‐Grissom et al. 2013). These contrasting differences might be caused by using different datasets.

It was noteworthy that Coenobitidae and Diogenidae shared a common ancestor, and these two families were closely clustered on the phylogenetic tree (Mclaughlin et al. 2007; Tsang et al. 2008). Based on our research, it can be observed that Coenobitidae diverged from Diogenidae and progressively evolved from a marine ancestor to adapt to terrestrial habitats, ultimately developing a high degree of terrestrial adaptability (~53 MYA). According to the divergence times, the terrestrial Coenobitidae transitioned from marine ancestors approximately 9.5–0.5 MYA. The period from 9.5 MYA to 0.5 MYA spanned the late Eocene to the early Pleistocene of the Cenozoic Era. During the late Eocene, the Earth's climate was relatively warm, which may have fostered an increase in biodiversity and an expansion of distribution ranges (Herman et al. 2017). We speculated that the warm climate during this climatic optimum could have provided hermit crabs with a broader range of habitats, facilitating their dispersal and diversification from marine to terrestrial environments. Throughout the Eocene, significant fluctuations in sea levels occurred, impacting the position of coastlines (De Lira Mota et al. 2023), which may have compelled hermit crabs to adapt to new terrestrial settings or recolonize marine environments during periods of sea‐level regression.

The transition to terrestrial environments has brought challenges to Coenobita, including water resource conservation, exposure to ultraviolet radiation, and oxygen availability (Nybakken and Bertness 2004). These selective pressures drove mitochondrial gene rearrangements mediated by mechanisms including tandem duplication–random loss (TDRL), transpositions, and inversions, with notable impacts on oxidative phosphorylation and antioxidant defense‐related genes. These genomic modifications likely reflect adaptive responses to terrestrial demands. In contrast, the stable marine habitat of Calcinus has maintained a more conserved mitochondrial gene arrangement (Mueller and Boore 2005).

The family Coenobitidae is recognized as the most terrestrial group in Anomura, having developed adaptations similar to those of terrestrial brachyurans, allowing them to effectively molt, mate, and spawn on land (Greenaway 2003). Most species within Coenobitidae are distributed in coastal and intertidal environments of tropical and subtropical regions. Compared to the stable marine environment, the intertidal zone is subjected to a multitude of stressors, such as the risk of drying out, exposure to intense UV rays, oxygen depletion, and variable salinity levels (Nybakken and Bertness 2004). Mitochondria may employ terrestrial adaptation strategies to facilitate the survival of fauna in this dynamic zone.

The mitochondrial genome of Coenobitidae exhibits positive selection pressure on the genes *ATP6*, *ND3*, *ND2*, *ND5*, and *ND6*, which encode components of Complex I and Complex V. These complexes play pivotal roles in the OXPHOS pathway, a central process in cellular energy metabolism (Trumpower 1990). We propose that positive selection on these genes enhances OXPHOS efficiency, elevating energy metabolism in Coenobitidae.

Beyond energy demands, terrestrial colonization also exposed Coenobitidae to elevated oxidative stress from UV radiation. During the process of energy metabolism, mitochondria generate reactive oxygen species (ROS), and ultraviolet radiation is another source of ROS (Gómez et al. 2023). The intertidal zone has a limited ability to filter UV radiation. High‐intensity UV rays could damage marine ancestors when they invade the intertidal zone (Cadet et al. 2005). ROS can pose a significant threat to cellular health (Jena et al. 2023). We proposed that the mitochondrial antioxidant system, in combination with the proteins encoded by the genes *ATP6*, *ND3*, *ND2*, *ND5*, and *ND6*, collectively maintains the redox balance within the cell (Rabilloud et al. 2001). It is posited that positive selection pressures may drive the evolution of these gene‐encoded proteins, conferring upon them enhanced antioxidant capabilities, thereby protecting the mitochondria from oxidative stress induced by ROS.

## 5 Conclusion

Our study provides the first complete mitochondrial genome sequences for five species from the families Coenobitidae and Diogenidae ( *C. elegans* , *C. gaimardii* , *C. latens* , *C. purpureus*, and *C. violascens*), which are essential data for clarifying terrestrial adaptation mechanisms within Anomura. The research conducted phylogenetic analyses and divergence times that trace the origins and evolutionary pathways of Anomura. It further confirmed that Paguroidea was paraphyletic and suggested that a more profound discussion of its internal relationships is necessary. Based on the study's findings, it is posited that Coenobitidae diverged from the aquatic Diogenidae and gradually evolved to terrestrial life through a series of genetic rearrangements, positive selection pressures, and other evolutionary processes. These discoveries are anticipated to aid in further phylogenetic reconciliation and to shed light on the evolutionary history of Anomura.

## Author Contributions

**Zhengfei Wang:** conceptualization (equal), data curation (equal), formal analysis (equal), funding acquisition (equal), methodology (equal), validation (equal), visualization (equal), writing – original draft (equal), writing – review and editing (equal). **Zhixuan Wang:** data curation (equal), formal analysis (equal), methodology (equal), software (equal), validation (equal), visualization (equal), writing – original draft (equal), writing – review and editing (equal). **Xin Chen:** formal analysis (equal), investigation (equal), methodology (equal), software (equal), visualization (equal), writing – original draft (equal). **Weijie Jiang:** formal analysis (equal), investigation (equal), software (equal), visualization (equal). **Chong Cui:** supervision (equal), writing – review and editing (equal). **Lijie Cui:** writing – review and editing (equal).

## Ethics Statement

Our research focuses on several common hermit crab species in nature, including *Calcinus elegans* , *Calcinus gaimardii* , *Calcinus latens* , *Coenobita purpureus*, and *Coenobita violascens*. While previous studies on related species have not raised animal ethics concerns, we strictly followed the ethical guidelines for animal welfare throughout our experiments. All sample collection and handling procedures complied with the Guidelines for the Review of Animal Welfare and Ethics in China, issued by the Animal Ethics and Welfare Committee of the Chinese Association for Laboratory Animal Sciences (CALAS).

## Conflicts of Interest

The authors declare no conflicts of interest.

## Supporting information


**Figure S1:** The map of the sampling area.

**Figure S2:** TheAT content of mitogenome and CR in five species.

**Figure S3:** RSCU of five species: *Calcinus elegans* (A), *Calcinus gaimardii* (B), *Calcinus latens* (C), *Coenobita purpureus* (D) and *Coenobita violascens* (E).

**Figure S4:** Inferred secondary structures of 22 tRNAs from five species ( *Calcinus elegans* , *Calcinus gaimardii* , *Calcinus latens* , *Coenobita purpureus*, and *Coenobita violascens*).

**Figure S5:** Gene rearrangement among Anomura crabs.

**Table S1:** The universal primers for two target genes.
**Table S2:** The GenBank accession numbers of 46 Anomura species and 1 Brachyura species in this study.
**Table S3:** Basic information on 3 fossil correction points.
**Table S4:** Mitogenome characteristics of five species of Anomura crab: *Calcinus elegans* , *Calcinus gaimardii* , *Calcinus latens* , *Coenobita purpureus* and *Coenobita violascens*.
**Table S5:** The nucleotide composition is based on five new species of mitogenomes containing A + T content of genes, AT‐skew, and GC‐skew.
**Table S6:** Table of AT content of 13 PCGs from 51 species.
**Table S7:** Table of GC content of 13 PCGs from 51 species.
**Table S8:** Genetic distances between 15 families and genetic distance within the Coenobitidae and Diogenidae.
**Table S9:** Rearrangement table, yellow and cyan genes color indicate they are coded on the two different strands.
**Table S10:** CODEML analyses of selection on mitochondrial genes in Anomura crabs.

## Data Availability

The data that support this study are available from the NCBI and are provided in Table S2.
