# Supplementary figures and images for: Based on Mitochondrial Genomes and Gene Order Rearrangements: Phylogenetic Relationships and Terrestrial Adaptability in Paguroidea (Crustacea: Decapoda)
Source: Ecol Evol. 2025 Aug 8;15(8):e71975. doi: 10.1002/ece3.71975 (PMC12334547; doi:10.1002/ece3.71975)

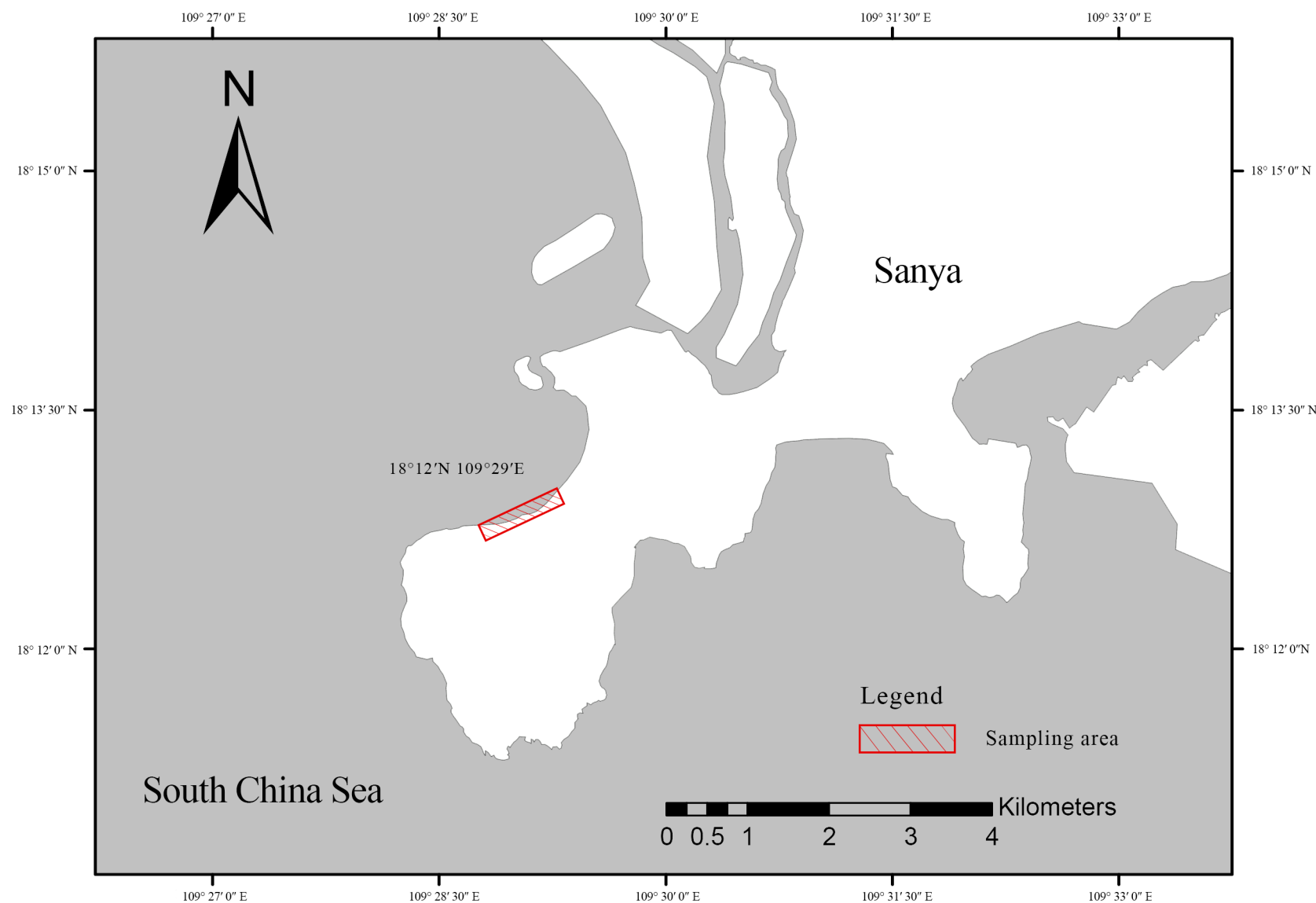

Supplement: Supplementary file 1 — Figure S1: The map of the sampling area. [file ECE3-15-e71975-s003.pdf]

A

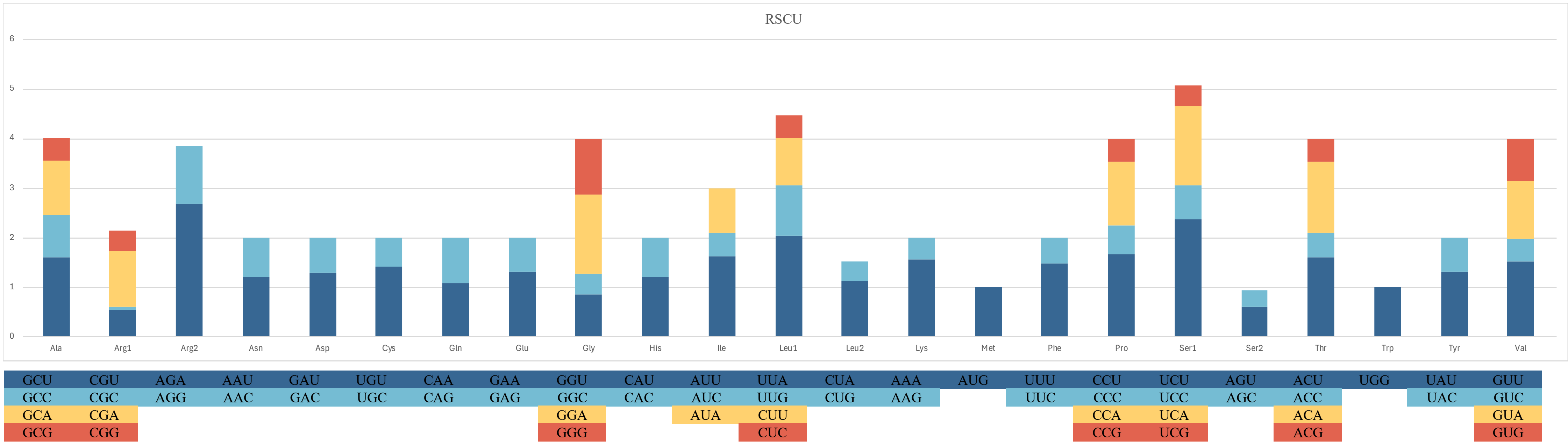

B

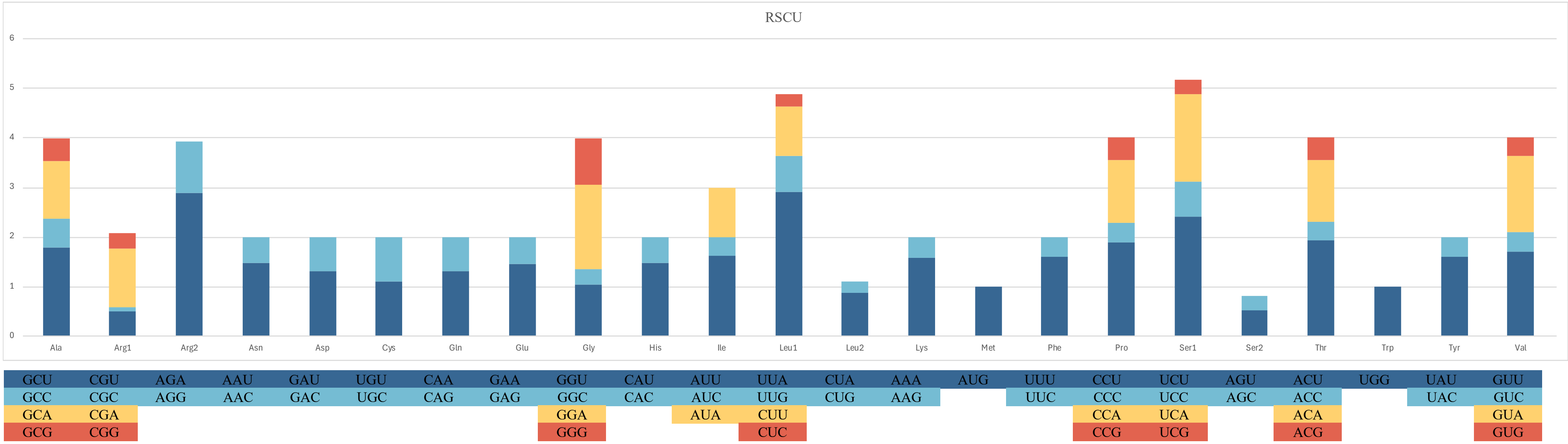

C

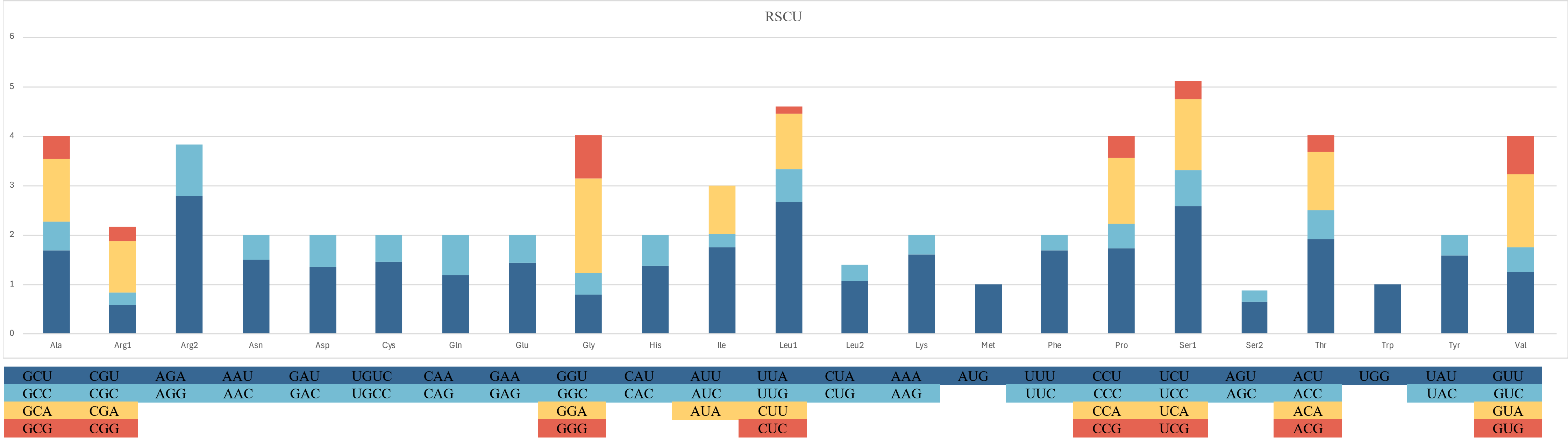

D

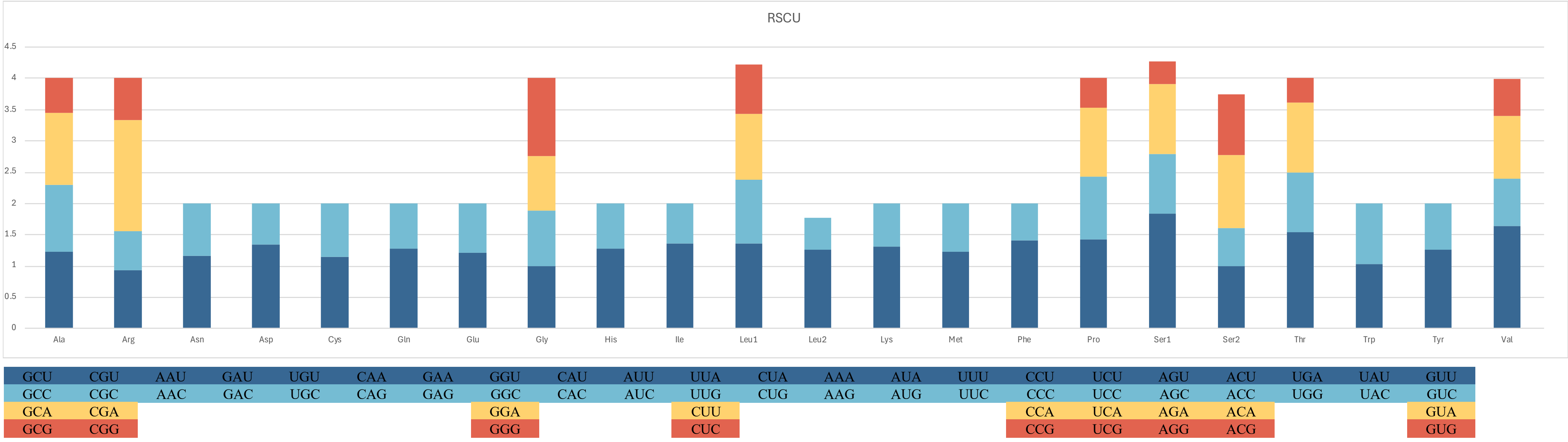

E

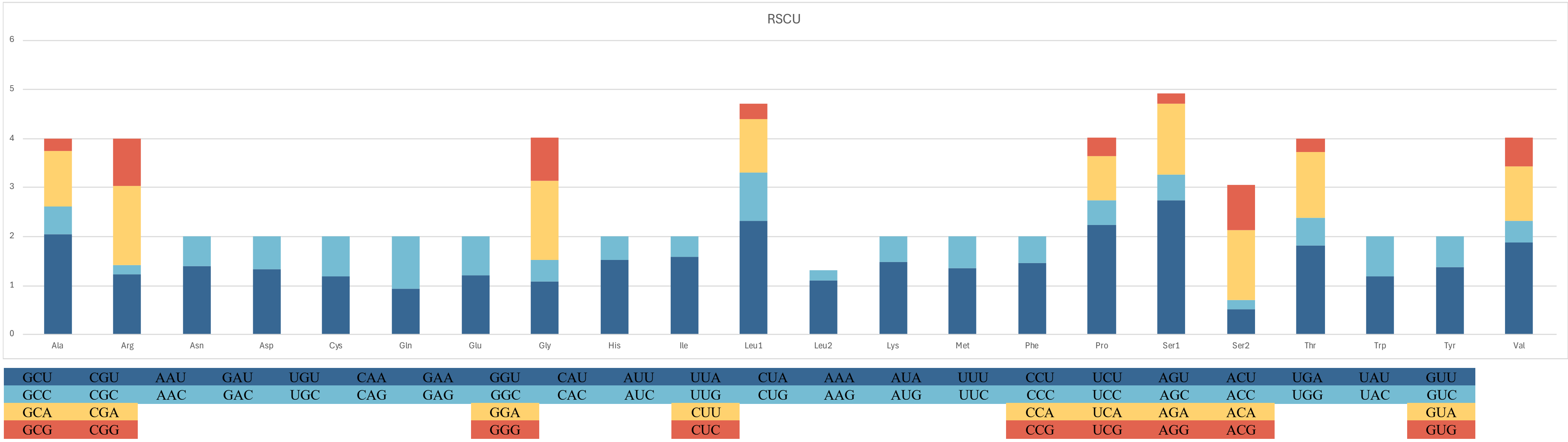

Supplement: Supplementary file 3 — Figure S3: RSCU of five species: Calcinus elegans (A), Calcinus gaimardii (B), Calcinus latens (C), Coenobita purpureus (D) and Coenobita violascens (E). [file ECE3-15-e71975-s001.pdf]

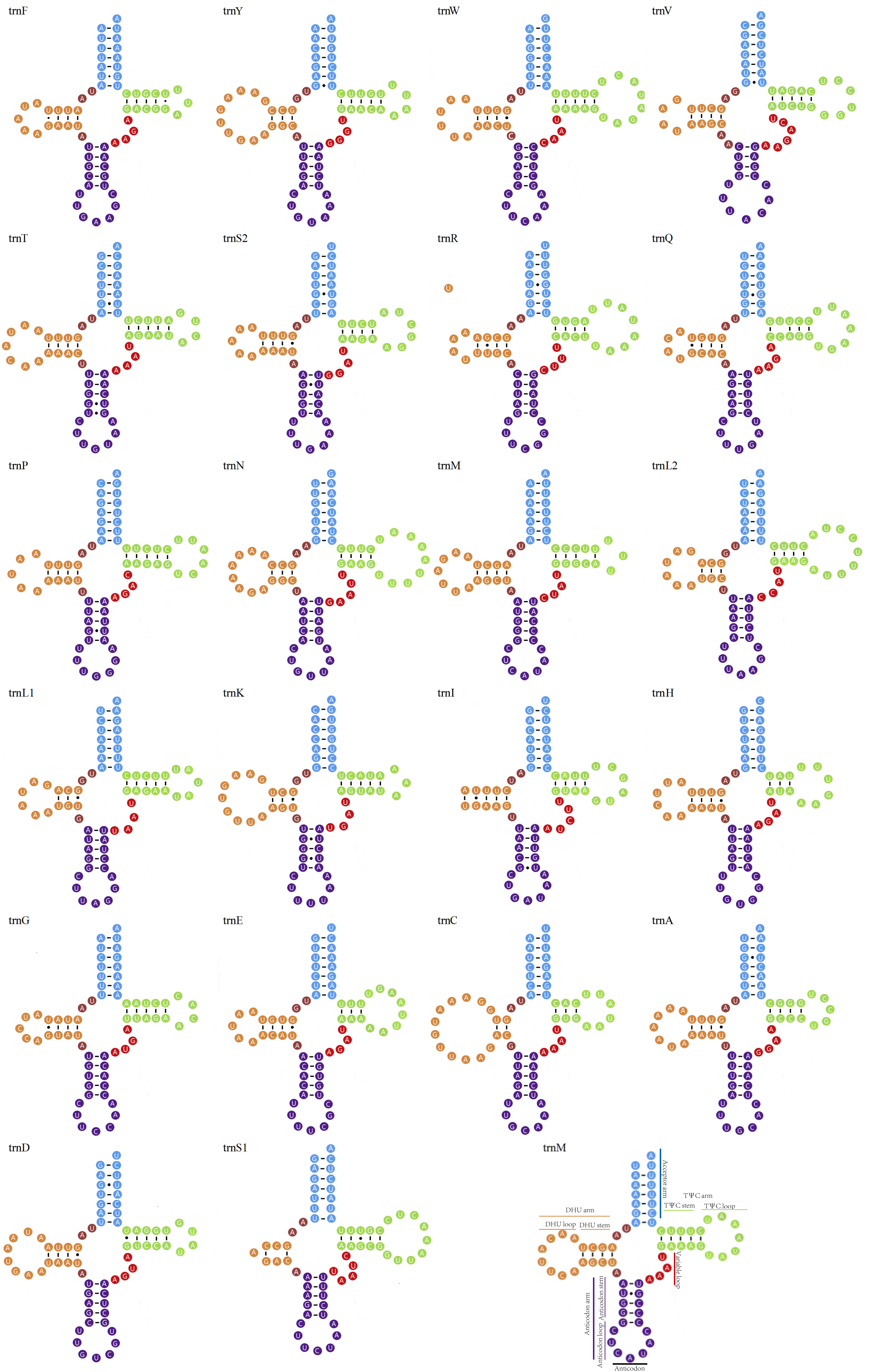

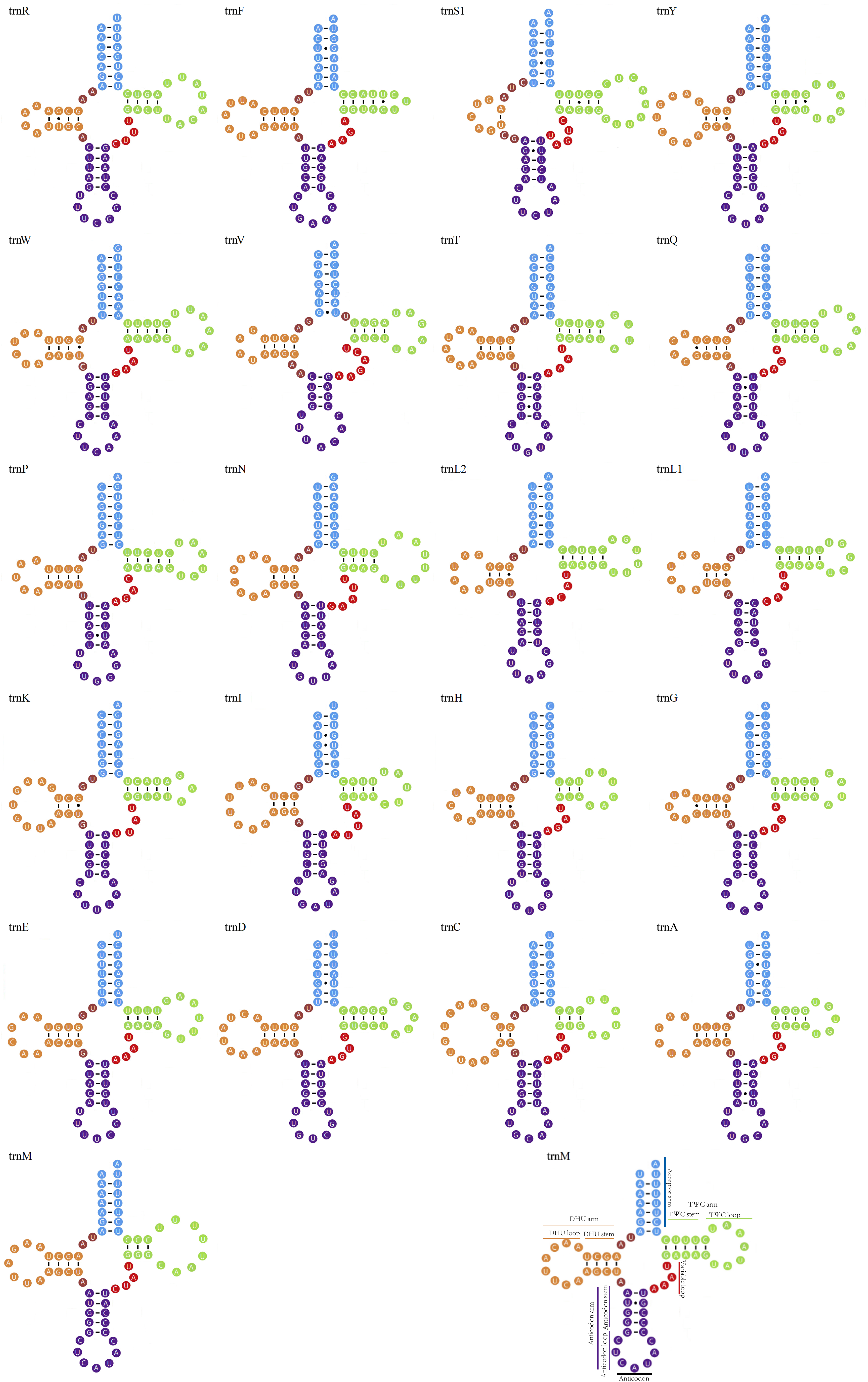

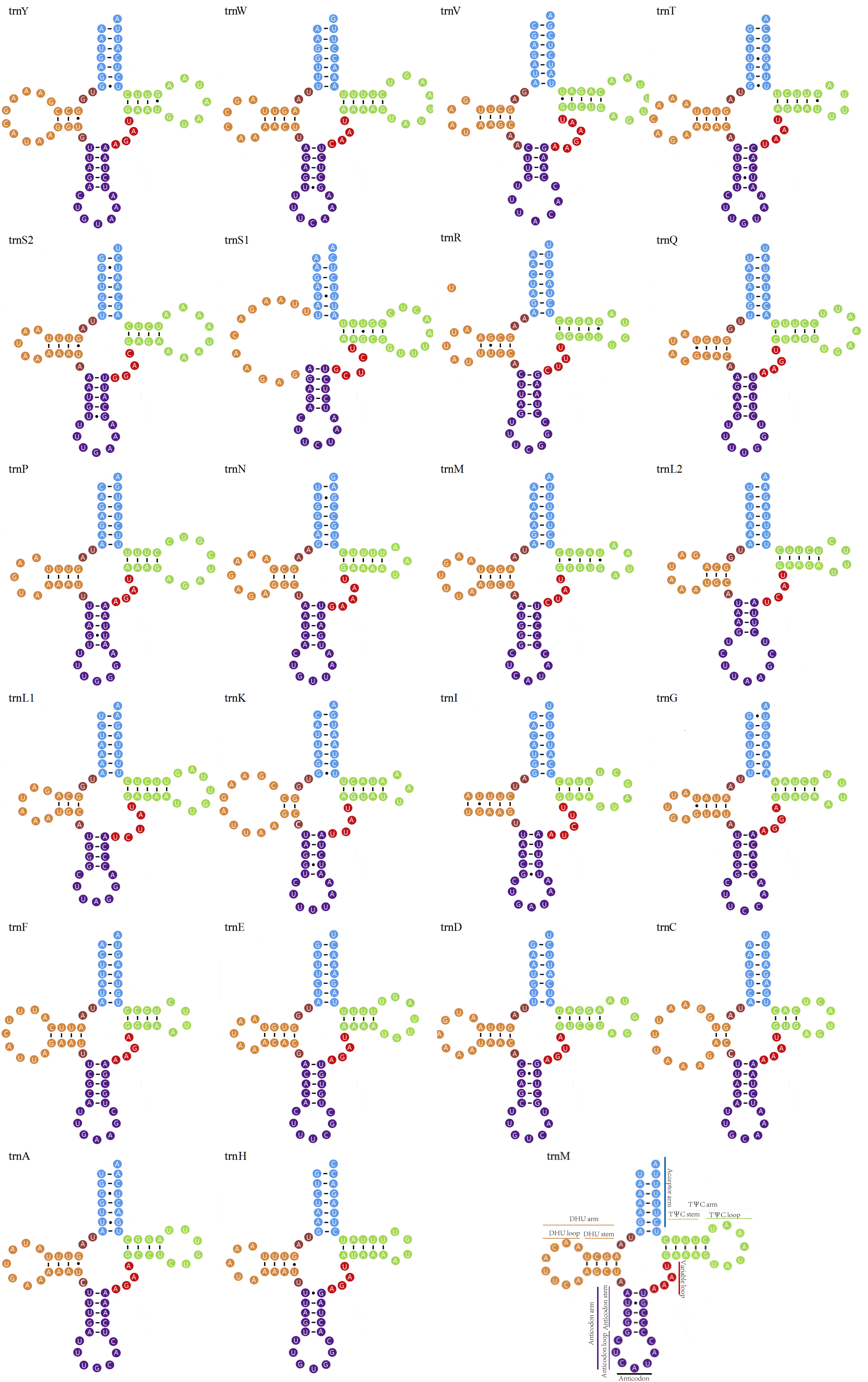

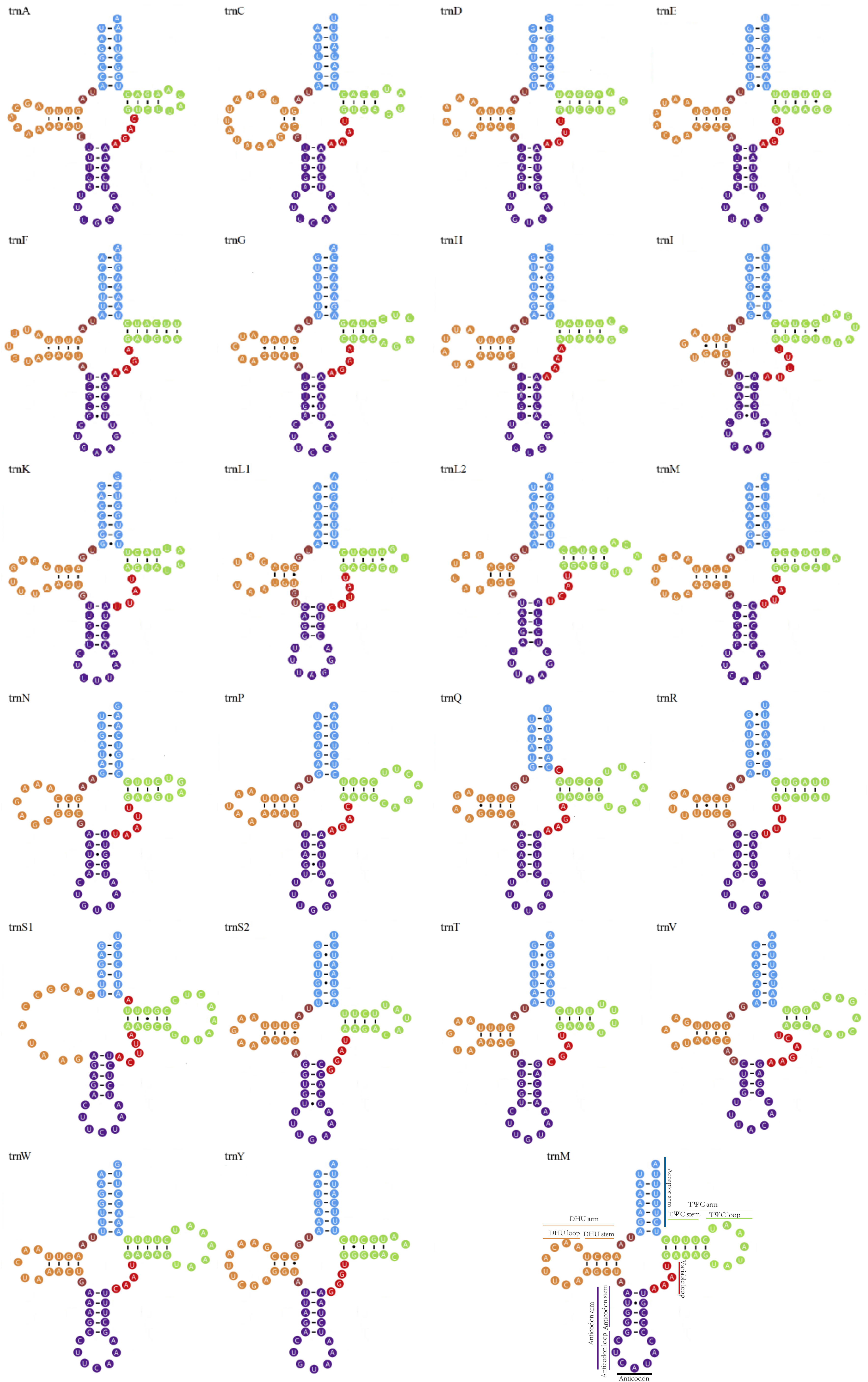

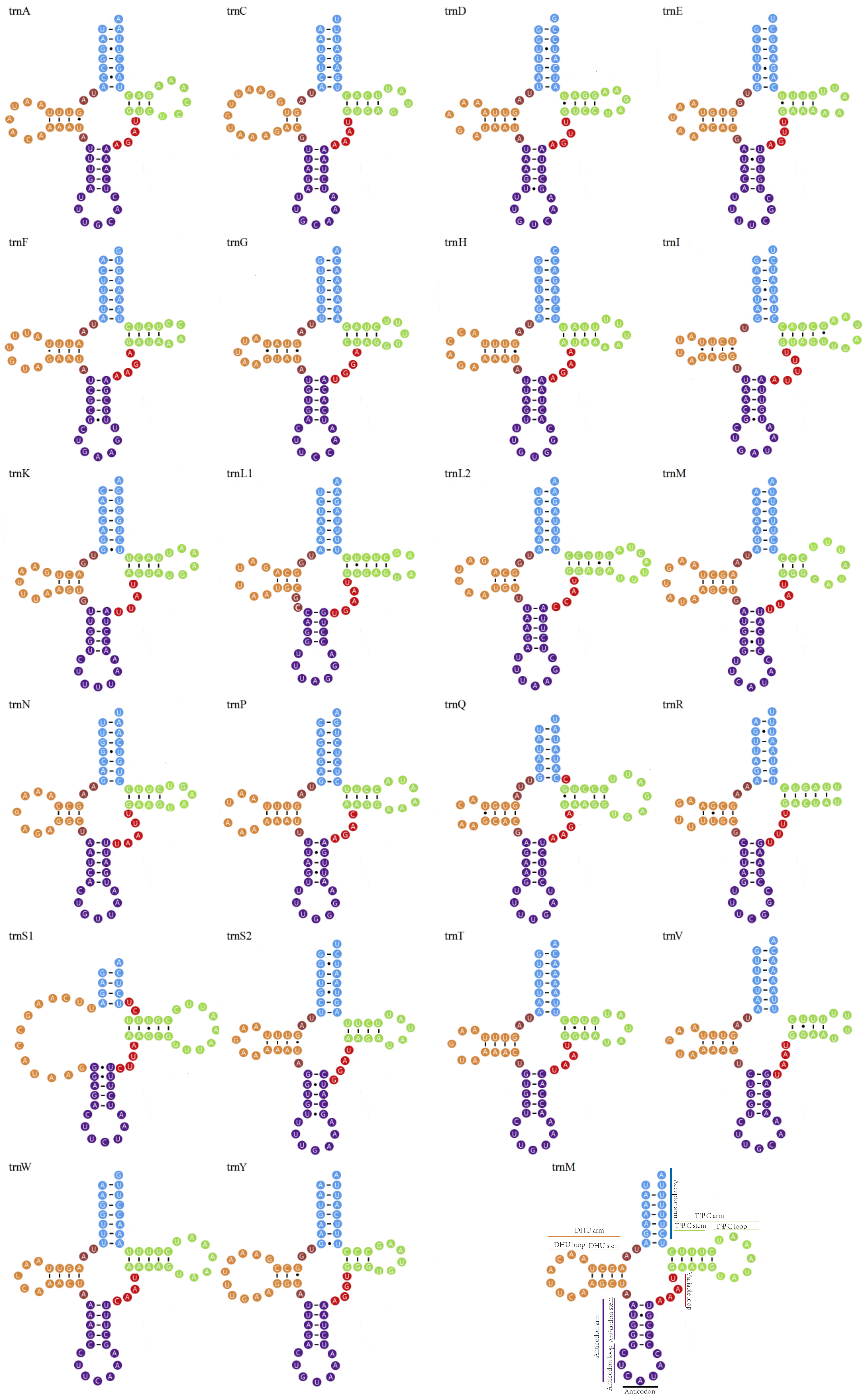

Supplement: Supplementary file 4 — Figure S4: Inferred secondary structures of 22 tRNAs from five species ( Calcinus elegans , Calcinus gaimardii , Calcinus latens , Coenobita purpureus, and Coenobita violascens). [file ECE3-15-e71975-s002.pdf]

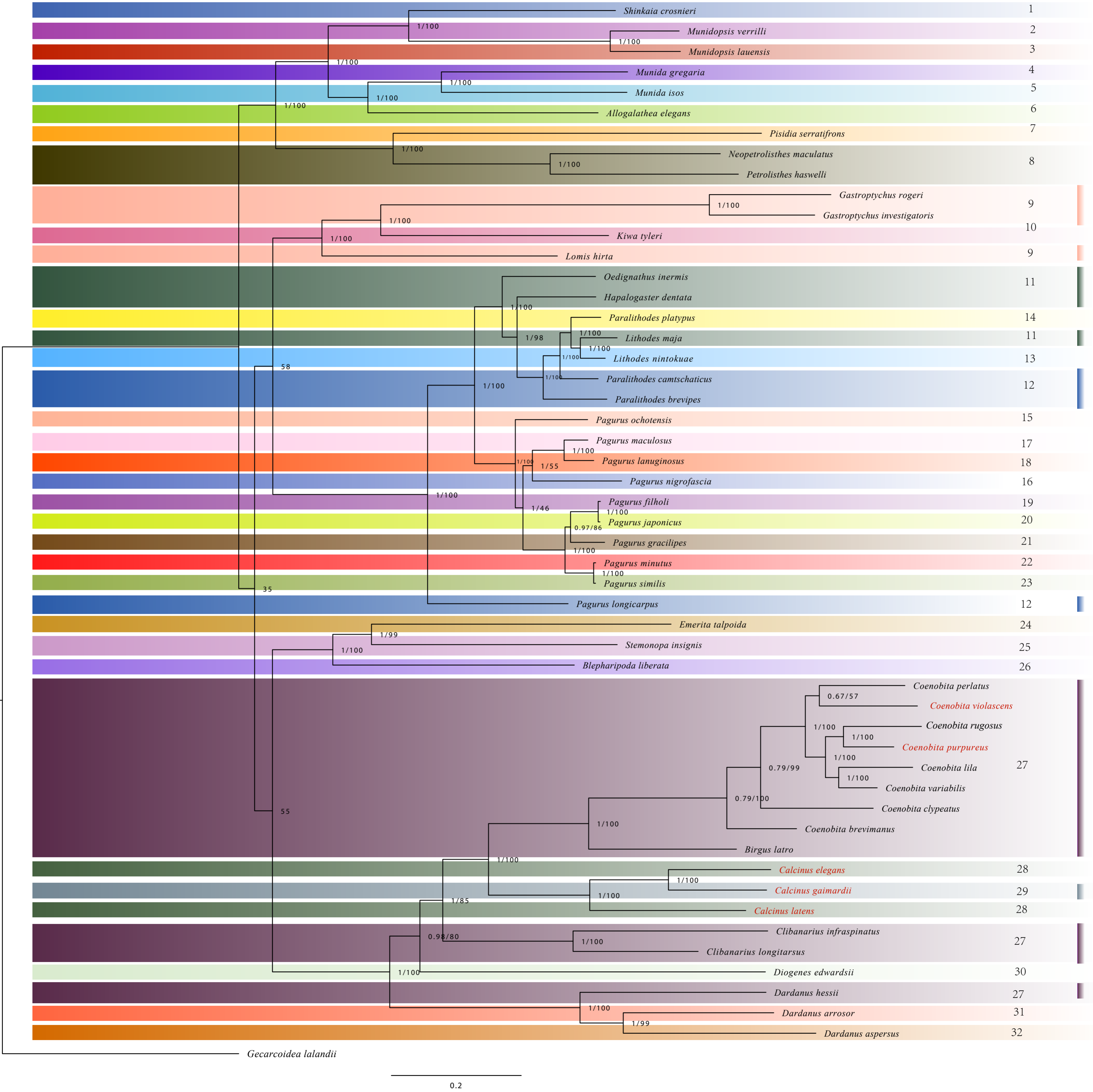

Supplement: Supplementary file 5 — Figure S5: Gene rearrangement among Anomura crabs. [file ECE3-15-e71975-s006.pdf]
