# Supplementary material for: Based on Mitochondrial Genomes and Gene Order Rearrangements: Phylogenetic Relationships and Terrestrial Adaptability in Paguroidea (Crustacea: Decapoda)
Source: Ecol Evol. 2025 Aug 8;15(8):e71975. doi: 10.1002/ece3.71975 (PMC12334547; doi:10.1002/ece3.71975)
Supplement: Supplementary file 2 — Figure S2: TheAT content of mitogenome and CR in five species. [file ECE3-15-e71975-s004.pdf]

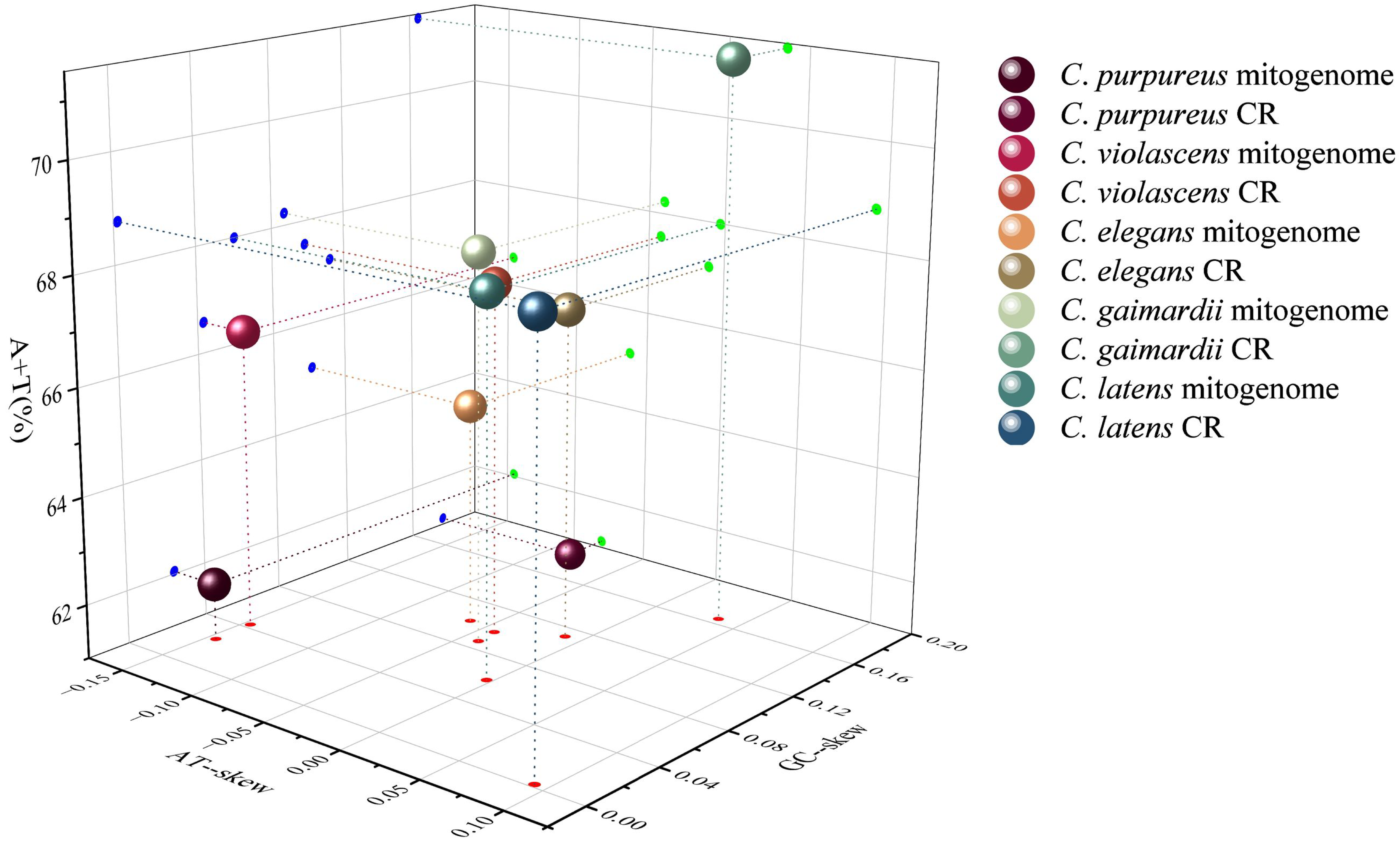

A+T(%)
62
64
66
68
70
AT--skew
−0.15
−0.10
−0.05
0.00
0.05
0.10
GC--skew
0.00
0.04
0.08
0.12
0.16
0.20
C. purpureus mitogenome
C. purpureus CR
C. violascens mitogenome
C. violascens CR
C. elegans mitogenome
C. elegans CR
C. gaimardii mitogenome
C. gaimardii CR
C. latens mitogenome
C. latens CR
